# Weight‐Length Ratio of Piranhas *Serrasalmus* (Characiformes, Serrasalmidae) in Bolivia: Relationships to Molecular Divergence and Maximum Size?

**DOI:** 10.1002/ece3.70970

**Published:** 2025-10-07

**Authors:** Fernando M. Carvajal‐Vallejos, Flavio Gallo‐Cardozo, Matías Careaga, Melina Campero

**Affiliations:** ^1^ Unidad de Limnología y Recursos Acuáticos (ULRA), Departamento de Biología, Facultad de Ciencias y Tecnología (FCyT) Universidad Mayor de San Simón (UMSS) Cochabamba Bolivia; ^2^ Museo de Historia Natural Alcide d'Orbigny Cochabamba Bolivia

**Keywords:** allometry, body size, Bolivian Amazon, fish biology, Palometa

## Abstract

Weight‐Length Relationships (WLRs) provide a basis for comparing life history strategies and morphological differentiation among fish species, potentially linking slope variations to evolutionary divergences in size and weight. This study presents the WLRs of nine *Serrasalmus* piranha species from the Amazon and La Plata basins in Bolivia, assessing whether WLRs slopes are influenced by phylogenetic relationships using a phylogenetic mixed model analysis on the mitochondrial DNA COI (barcoding) locus. All species demonstrated an exponential (power‐type) growth pattern, with most showing positive allometric growth. The species showing the greatest differentiation in both WLRs and genetic variation was *S. elongatus* . We detected a strong phylogenetic signal in WLR slopes, though clustering techniques for WLRs slopes and molecular data revealed only partial concordance. We discuss how these concordances and discrepancies between WLRs and genetic data may reflect ancient and intermediate speciation events, shaped by habitat conditions and stochastic evolutionary processes. Such processes appear to influence swimming mechanisms and ecological niche navigation in these closely related *Serrasalmus* species.

## Graphical abstract

This study presents WLRs of nine piranha or palometa fish species of genus *Serrasalmus* present in the Amazon and La Plata basins of Bolivia and compares differences with genetic divergence (mtDNA COI locus) and the observed maximum size. All species showed an exponential (power type) growth pattern, and all WLRs were significantly different among all species. We detected a strong phylogenetic signal in WLRs slopes, though clustering techniques for WLRs slopes and molecular data revealed only partial concordance.

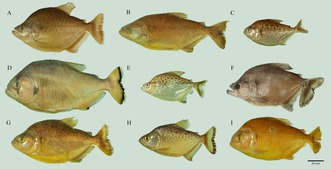

## 1 Introduction

Differential relative growth is defined as allometric growth (one type of ontogenetic variation) and involves a variation in shape related to variation in size (Lleonart et al. 2000; Osse and van den Boogaart 2004). During development, alterations in the body shape of fishes are related to the feeding and swimming modes (Russo et al. 2007; Dikou 2023). Thus, the length‐weight relationships (WLRs) differ among species and depend on inherited body shape and physiological factors such as energy allocation, maturity (life stage), spawning, and trophic level (Jisr et al. 2018; Dikou 2023).

WLRs are highly important in fisheries biology, as they help describe changes in the size of individuals, showing the growth pattern of fishes, obtaining the index of physical condition of populations and evaluate habitat quality (Gayon 2000; Albuquerque et al. 2009). WLRs are considered an important biological marker indicating the degree of well‐being or fatness of fishes (individuals of a given length that are heavier are considered to be in better condition) and can be used to obtain spatial and temporary yield of natural populations under fisheries management (i.e., fish crop biomass), and thereby base line data on stock conditions, among other biological information (Ragheb 2023; Kumary and Raj 2016). WLRs have been associated with body form and with energy consideration and have been suggested that concurrent stunting and starvation are generated at marginal values of the *a* (coefficient of body form) and *b* (exponent associated with the type of fish growth, reflects isometry when the value equals 3) parameters (*W* = a*L ^b^), the latter being considered an accelerator, and the former a brake, in the correspondence between fish condition and environmental conditions (Dikou 2023).

Among the wide range of applications of WLRs (Karachle and Stergiou 2012), it allows comparisons among functional ecological strategies (i.e., trophic ecology related to ontogeny and feeding patterns related to size—de Almeida et al. 1998; Agostinho et al. 2003; Ferreira et al. 2014), life history strategies, and morphologic differentiations of the same species in different areas or among species (Froese and Binohlan 2005; Li et al. 2023). It has been proposed that herbivorous fish tend to be shorter and flatter compared to carnivorous fish, with omnivorous having characteristics that are intermediate (Blake 2004; Akin and Winemiller 2008). Fishes living in lentic waters tend to have a shorter and flatter body shape compared to those living in lotic waters (Li et al. 2023). On the other hand, non‐migratory or short‐distance migratory ray‐finned fish species evolved shorter body length than migratory relatives (Burns and Bloom 2020; Li et al. 2023), showing an adaptive response in WLRs. Additionally, it is known that fishes have repeatedly transitioned between pelagic (limnetic/open water), demersal (close proximity to the substrate), and fully benthic (in physical contact with substrate) habitats, shifts that are thought to have substantial implications for the evolution of body form (Friedman et al. 2020). Habitat divergence promotes coexistence in many aquatic systems (i.e., Hollingsworth et al. 2013), and the ecomorphological axis of body shape changes is linked to transition patterns between pelagic and demersal habitats (Friedman et al. 2020). Therefore, microevolutionary divergence of fishes is normally driven by natural selection or genetic drift (Albert and Johnson 2012), which is greatly determined by the diversity and complexity of aquatic habitats, and body size diversity tends to accumulate along trajectories close to isometry (Alencar et al. 2022).

**FIGURE 1:**
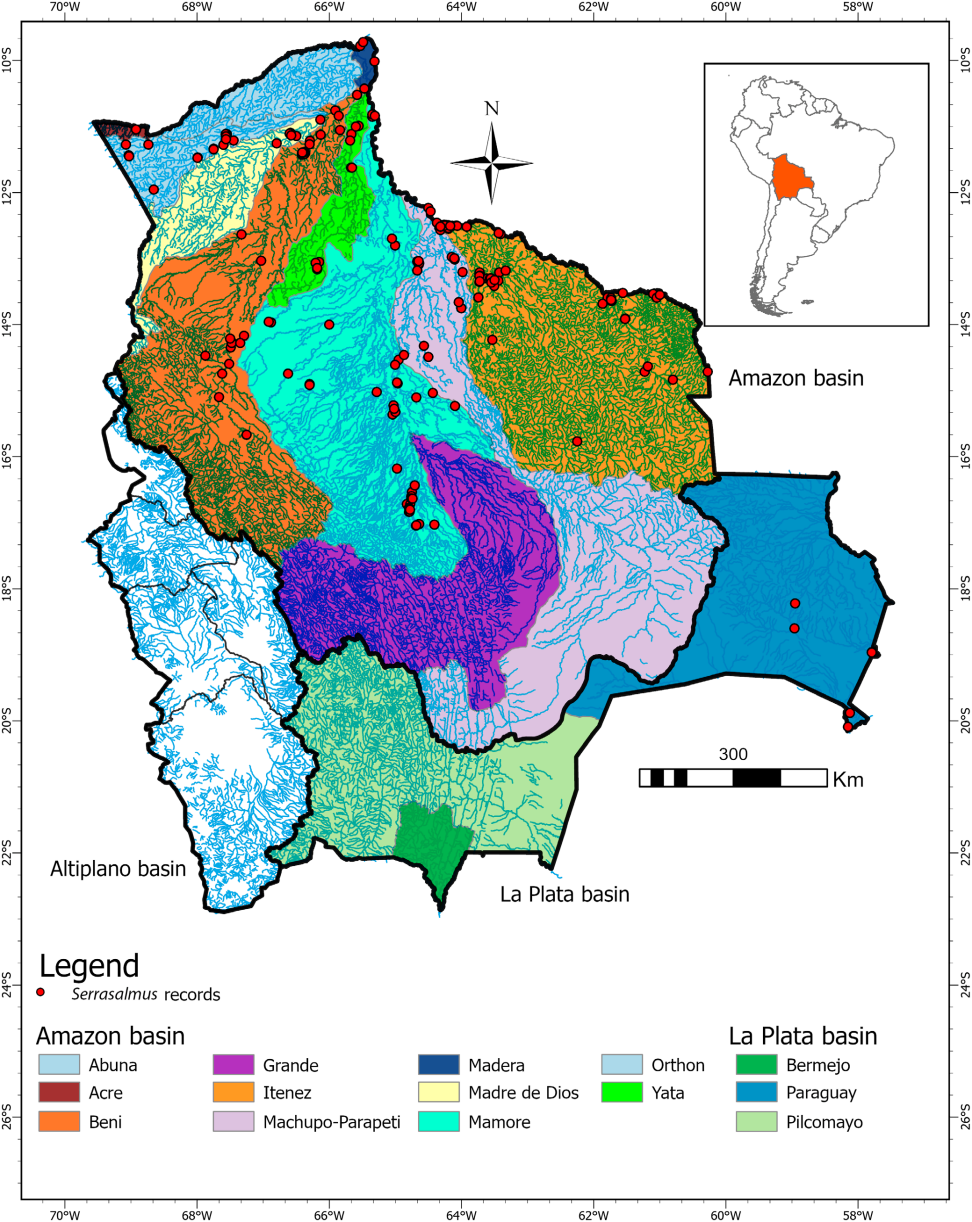
Map showing the origins of *Serrasalmus* samples in Bolivia, across the Amazon and La Plata basins and their respective sub‐basins within the country. Original elaboration created using ArcGIS Pro version 3.3.1 software.

Several responses of body size to environmental heterogeneity and connectivity are concordant for the Serrasalmids (Characiformes) fish species inhabiting rivers and lagoons in lowlands of the Neotropical region of South America. The big pacú and/or tambaquí of genera *Colossoma* and *Piaractus* (Colossomatinae) can attain more than 820 mm of standard length (SL) (> 23.0 kg) and 700 mm SL (> 14.0 kg), respectively (Loubens and Aquim 1986; Issac and Ruffino 1996; Loubens and Panfili 1997, 2001; Maldonado 2004), and undertake periodic long‐distance migrations (~100–1000 km) for dispersal, reproduction, and/or feeding, traveling toward tributaries in the headwaters of the Amazon, Orinoco, and La Plata basins (Araujo‐Lima and Goulding 1997; Calcagnotto and DeSalle 2009; Van Damme et al. 2011). On the other hand, the related species of the piranha genera *Serrasalmus* and *Pygocentrus* (Serrasalminae), which are medium to small in size (< 300 mm SL, < 1.0 kg), are considered resident or short‐distance migratory species (< 100 km) (Agostinho et al. 2004, 2007; Van Damme et al. 2011; Makrakis et al. 2012). In this study, nine piranha species of the genus *Serrasalmus*, from 32 valid species (morphologically identifiable and recognized in scientific publications and fish catalogs) (Fricke et al. 2023; Gallo‐Cardozo et al. 2024), were recorded in different sub‐basins from the Amazon and La Plata basins of Bolivia (Carvajal‐Vallejos and Zeballos 2011; Carvajal‐Vallejos et al. 2014; Gallo‐Cardozo et al. 2024) (Figure 1). Although morphological differentiation among these species is more pronounced, genetic divergence is less evident among some of them, likely due to recent speciation events within the same watershed (Hubert et al. 2008; Gallo‐Cardozo et al. 2024). Additionally, introgression has been proposed to occur among some species (Hubert et al. 2008), obscuring the evolutionary forces responsible for their shared morphological and genetic polymorphism. Bolivian species are primarily carnivorous (Ayala et al. 2000) inhabiting lagoons, streams, and rivers of white (high content of suspended sediments) and clear waters (low content of suspended sediments) (Carvajal‐Vallejos et al. 2014). It was shown that *S. rhombeus* and *S. magallanesi* (previously misidentified as *S. humeralis* ) are strict piscivores (whole fish, muscle fragments, bitten‐off fins), while *S. elongatus* exhibits more generalist carnivorous feeding habits, consuming fish as well as other items such as aquatic and terrestrial insects, decapods, and a minor proportion of plant material (Ayala et al. 2000). Although no information is available on the diet of *S. maculatus* in Bolivia, studies in Brazil indicate that fin‐nipping on other fish is an important contribution to the diet of this species among other items (i.e., fish whole or muscle tissue, insects, and plant remnants), making it more akin to parasitism than predation (Agostinho et al. 2003; da Silva et al. 2015). Most of the Amazonian species, if not all, coexist in the same water bodies (sympatry) or adjacent habitats (Carvajal and Maldonado 2005; Maldonado and Carvajal 2005). It has been suggested that niche partitioning (different use of resources) and seasonality (temporal availability of prey or food) may enable their coexistence in lagoons, though occasional cannibalism does occur (De Andrade et al. 2024). Life history traits are unknown in Bolivian territory but it is known from other areas that they are preferably *r*_2_ (periodic) and *r*_1_ (opportunistic) strategists (Winemiller and Taphorn 1990). Some species, as *S. marginatus* , are solitary, but others, as *S. spilopleura* , live in shoals (Sazima and Machado 1990). Based on their rarity or notable abundance in captures conducted in the Bolivian Amazon, certain species such as *S. elongatus* , *S. compressus* , and *S. magallanesi*, could be considered solitary. In contrast, others, like *S. rhombeus* , *S. maculatus* , and *S. odyssei*, seem to live in shoals. *Serrasalmus* species commonly known as “piraña” or palometa contribute significantly to food security in both small indigenous towns and big urban towns through subsistence and commercial fisheries in Bolivia (Montellano et al. 2017). WLRs and maximum sizes of these piranha species in Bolivia are still unknown, as well as whether their relationships among WLRs could be related to macro‐ or microevolutionary size‐weight divergence among species.

The aim of the present study was to determine the weight‐length relationships (WLRs) of nine piranha species present in the lowlands of Bolivia and to assess whether these relationships, along with the observed maximum standard length (MSL) of each species (a body‐form trait), align with the phylogenetic divergence within the group, as measured using the barcoding locus COI mtDNA. Based on this aim, the hypotheses tested were: (a) The WLRs and maximum size of the piranha species are influenced by aquatic environmental conditions and interspecific ecological interactions; (b) Species with similar WLRs and maximum size will exhibit greater molecular similarity, reflecting phylogenetic relatedness.

## 2 Materials and Methods

### 2.1 Data Collection

Morphological and genetic data were obtained from different sets, but all individuals with available sequences in BOLD (Barcode of Life Data System) were measured (Table 1). A total of 224 individuals of nine species of piranhas *Serrasalmus* from Bolivia (Figure 1.) were analyzed; 220 housed in the ichthyological collection of Natural History Museum Alcide d'Orbigny—Unidad de Limnología y Recursos Acuáticos (ULRA), University Mayor de San Simón (UMSS) (Cochabamba, Bolivia), and four in the Centro de Recursos Acuáticos, University Autónoma del Beni José Ballivián, Trinidad (CIRA‐UAB‐JB, Beni, Bolivia) (Table S1).

**TABLE 1:** List of analyzed species of piranhas *Serrasalmus* from the Amazon and La Plata basins of Bolivia.

Species	BOLD ID	GenBank ID	WLRs/MSL
*Serrasalmus compressus*	BBF168‐13	—	27
*Serrsalmus hollandi*	BBF194‐13	—	49
*Serrasalmus rhombeus*	BBF179‐13	—	23
*Serrsalmus magallanesi*	BBF173‐13	—	29
*Serrsalmus maculatus*	BBF158‐13	KP256377.1	22
*Serrsalmus elongatus*	—	MG752619.1, MG752622.1	20
*Serrasalmus spilopleura*	—	MG752822.1, MG752826.1	7
*Serrsalmus marginatus*	—	KP256399.1	24
*Serrasalmus odyssei*	—	—	23

*Note:* BOLD ID shows code identification in BOLD System of COI mtDNA haplotypes obtained for Bolivian specimens. GenBank ID shows code identification of haplotypes available in National Center for Biotechnology Information (NCBI) (1988). WLRs/MSL displays the number of specimens per species for which Weight‐Length relationships and maximum standard length was obtained.

Abbreviation: —, Missing data.

**FIGURE 2:**
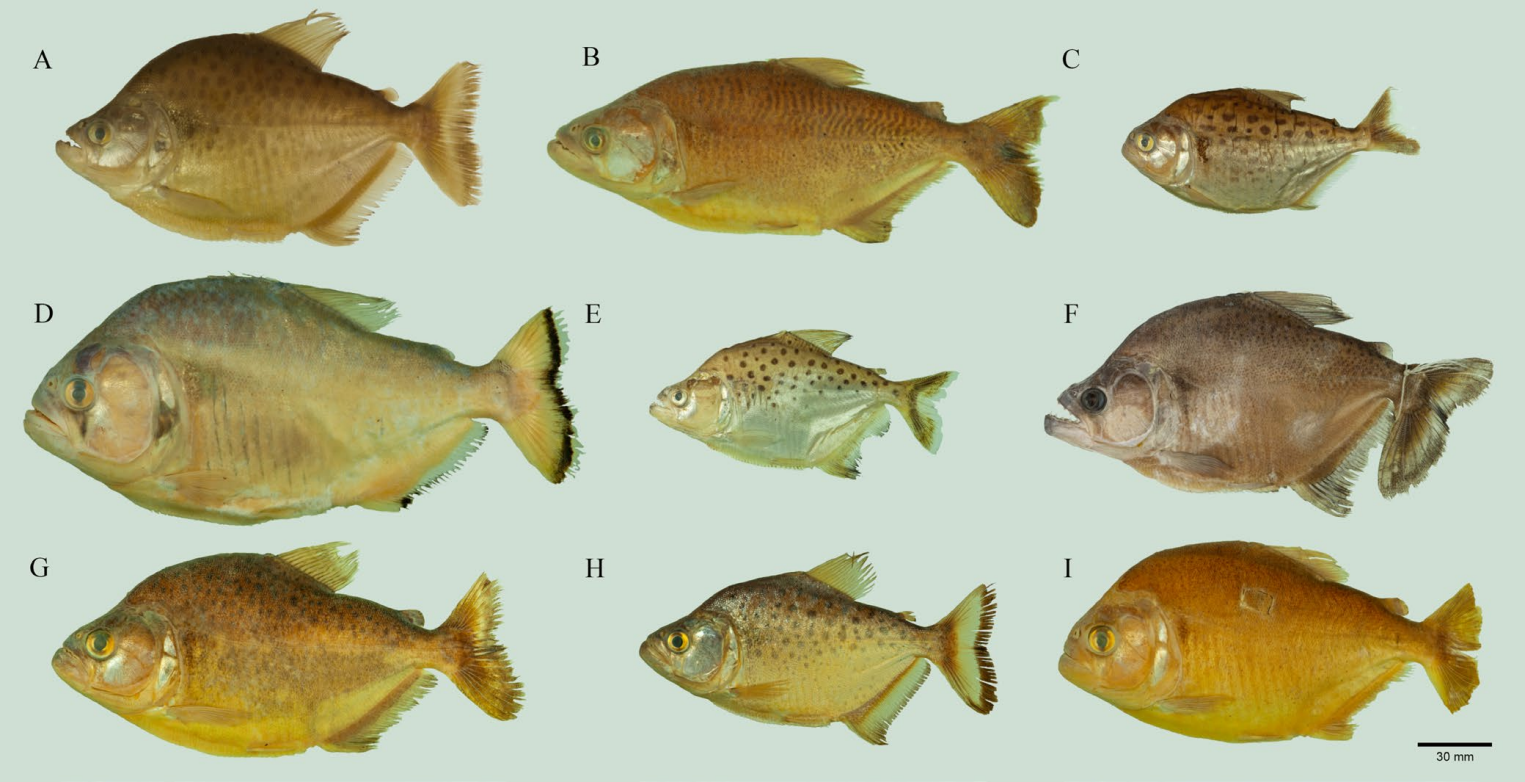
Plate of Bolivian species of *Serrasalmus* species and lot number. (A) *S. compressus* , UMSS 1912; (B) *S. elongatus* , UMSS 12201; (C) *S. hollandi* , UMSS 12287; (D) *S. maculatus* , UMSS 12195; (E) *S. magallanesi*, UMSS 11171; (F) *S. marginatus* , UMSS 12258.1; (G) *S. odyssei*, UMSS 4538; (H) *S. rhombeus* , UMSS 12311.2; (I) *S. spilopleura* , UMSS 1590.

27 specimens corresponded to *Serrasalmus compressus* Jégu et al. 1991, 20 to *Serrasalmus elongatus* Kner 1858, 49 to *Serrasalmus hollandi* Eigenmann 1915, 22 to *Serrasalmus maculatus* Kner 1858, 23 to *Serrasalmus odyssei* Hubert and Renno 2010, 23 to *Serrasalmus rhombeus* (Linnaeus 1766), 29 to *Serrasalmus magallanesi* Gallo‐Cardozo et al. 2024, seven to *Serrasalmus spilopleura* Kner 1858, from Amazon basin, and 24 to *Serrasalmus marginatus* Valenciennes 1837 from La Plata basin (Figure 2).

Standard length (SL—cm) was obtained using calipers (Clockwise Tools) with accuracy of 0.1 mm, and weight using a balance Ohaus SJX6201M with accuracy of 0.1 g.

### 2.2 Allometry

All analyses and graphs were performed in R‐studio (R‐project v4.3.1, R core team, 2024), with different packages that are mentioned in the following sections. The WLRs were graphically explored with the raw data, showing an exponential relationship (*r* > 0.79). To obtain the constant a value (related to body shape), and the type of allometry (*b*) of each species, the raw data was fitted with the $y=aX^{b}$ equation.

### 2.3 Genetic Analysis

Genetic analysis was carried out on 16 haplotypes of the COI (Cytochrome Oxidase I ~ 578 bp) mtDNA locus belonging to eight species of piranhas *Serrasalmus* and four external groups. *Serrasalmus odyssei* was not included in the genetic analysis because it was not possible to collect muscle tissue for sequencing analyses, and no sequences were publicly available. Sequences of Bolivian specimens were obtained in the Canadian Center of Barcoding (Guelph, Canada) deposited in Barcode of Life Data Systems (BOLD) (www.barcodinglife.org), and other complementary sequences were obtained from National Center for Biotechnology Information (NCBI) (1988). GenBank sequences were published by Machado et al. (2018) and Bignotto et al. (2020).

Sequences of *S. compressus* , *S. hollandi* , *S. rhombeus* , *S*. *magallanesi*, and *S. maculatus* were obtained from Bolivian specimens and are deposited in Barcode of Life Data Systems (BOLD) (www.barcodinglife.org) as BBF168‐13, BBF169‐13, BBF194‐13, BBF195‐13, BBF179‐13, BBF173‐13, and BBF158‐13 (Table 1). Other sequences included in the analysis were those of *S. elongatus* from Brazil (MG752619.1 and MG752622.1—Machado et al. 2018), *S. spilopleura* from Brazil (MG752822.1 and MG752826.1—Machado et al. 2018), *S. maculatus* from Brazil (KP256377.1—Bignotto et al. 2020), and *S. marginatus* from Brazil (KP256399.1—Bignotto et al. 2020) (Table 1).

*Pygocentrus nattereri* Kner 1858, *Catoprion mento* (Cuvier 1819), *Piaractus brachypomus* (Cuvier 1818), and *Colossoma macropomum* (Cuvier 1816) (Serrasalmidae family) from Bolivia were included as external groups and are deposited in BOLD system as BBF 141–13, BBF 142–13, BBF 156–13, and BBF 161–13.

Sequence alignment was performed using the Muscle algorithm (Robert 2004) in the “msa” package (Bodenhofer et al. 2015). The best evolutionary model of nucleotide substitution was selected by using the “phangorn” package (Schliep et al. 2019) and decided according to the Bayesian information criterion (BIC). The maximum likelihood tree was obtained using the Hamming distance and Neighbor‐joining clustering in the “pegas” package (Paradis 2010), using the optimum nucleotide substitution model (HKY + G(4), shape parameter = 0.17). A test of phylogeny was carried out by running a bootstrap analysis of 100 replicates in the “ape” package (Paradis and Schliep 2019).

### 2.4 Weight–Length Relationships

To explore whether there were differences between WLRs among species, and given the fact that this was a comparison between closely related species, a phylogenetic linear mixed model (i.e., Gaussian distribution) was performed with the “phyr” package (Li et al. 2020). In order to linearize the WLRs, both variables (weight and standard length) were log‐transformed. A mixed model with weight as dependent variable, standard length as fixed factor, and species, species phylogenetic covariance and the interaction between species*standard length as random factors, was run. Model assumptions such as linear relationship between dependent and independent variables, variance homogeneity, and normal distribution of the errors were correctly fulfilled by our dataset. The significance of the random factors was tested by means of likelihood ratio tests (LRT). Because the random effect of the interaction between standard length and species was significant (see Results), to better visualize the results, a fixed model with standard length and species*standard length was also run. Finally, an adjusted‐Tukey pairwise comparison of slopes was performed and was graphically represented by a hierarchical cluster analysis of their Euclidean distances (UPGMA aggrupation method).

### 2.5 Genetic Divergence, WLRs, and MSL Differences

To further explore the relationship between genetic and WLRs slopes, a linear regression between both set of distances was performed. Additionally, because both the WLRs pattern and the maximum standard length (MSL) are heritable traits that could reflect time since divergence (DeLorenzo et al. 2023), the relationship between MSL, WLRs (expressed as Euclidean distances), and genetic divergence between species was explored via linear regression. Finally, the relationship between WLR and MSL was also obtained by linear regression.

## 3 Results

### 3.1 Allometry

**FIGURE 3:**
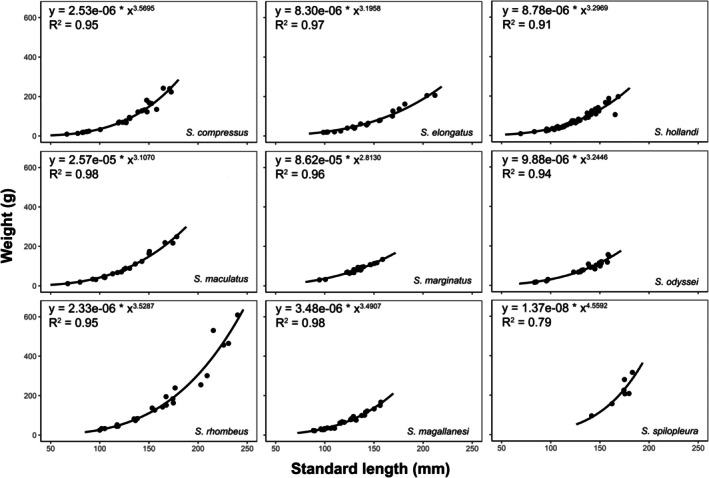
Length‐weight relationship and allometry (raw data) in nine piranha *Serrasalmus* species from Bolivia. Data fit $y=aX^{b}$.

All species showed an exponential (power type) growth pattern as depicted in Figure 3. As expected, based on Carlander's (1985) study, the parameter *b* was between 2.7 and 3.5. The only exception was *S. spilopleura* , with *b* = 4.56. From all species, *S. marginatus* was the only one that showed a *b* < 3 (2.7).

### 3.2 Genetic Analysis

**FIGURE 4:**
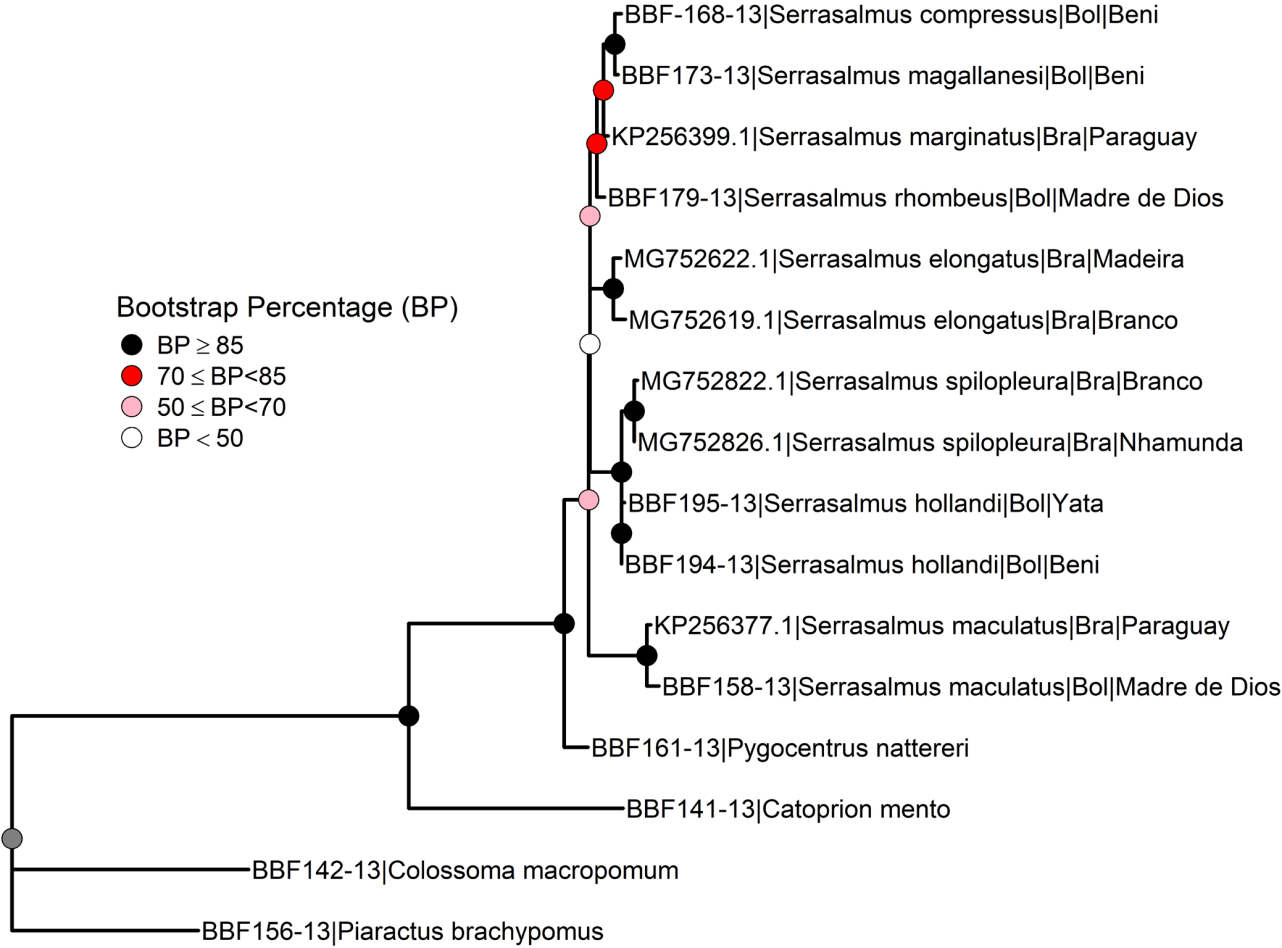
Maximum Likelihood tree (bootstrap 1000, model HKY + Gamma (0.17)) built on 16 haplotypes (mtDNA COI locus ~578 bp) of eight piranha *Serrasalmus* species from Bolivia. Bootstrap percentage in the legend. Bol, Bolivian origin; Bra, Brazilian origin.

Hasegawa‐Kishino‐Yano model + Gamma (0.17) was the best evolutionary model of nucleotide substitution. COI sequence showed low divergence (mean value less than 1%) among species *S. compressus* and *S. magallanesi* from Bolivia (Table 2). The ML tree showed that *S. rhombeus* , *S. marginatus* , *S. magallanesi*, and *S. compressus* (bootstrap 67) form a group of closely related species, *S. elongatus* (bootstrap 80) and *S. maculatus* (bootstrap 98) show to be the most differentiated species and *S. hollandi* and *S. spilopleura* (bootstrap 99) appear to be another set of closely related species (Figure 4).

**TABLE 2:** Genetic divergence and standard deviation at the mtDNA COI locus (578 bp) among eight species of piranha *Serrasalmus* species from Bolivia.

	*S.spi* (Bra)	*S.rho* (Bol)	*S.mar* (Bra)	*S.mag* (Bol)	*S.mac* (Bol)	*S.elo* (Bra)	*S.hol* (Bol)	*S.com* (Bol)
*S.spi*		0.012	0.011	0.015	0.017	0.012	0.004	0.016
*S.rho*	0.045		0.005	0.006	0.014	0.009	0.010	0.007
*S.mar*	0.037	0.012		0.005	0.016	0.010	0.008	0.006
*S.mag*	0.056	0.015	0.012		0.016	0.012	0.011	0.004
*S.mac*	0.070	0.056	0.060	0.065		0.013	0.015	0.014
*S.elo*	0.046	0.028	0.030	0.043	0.057		0.011	0.012
*S.hol*	0.009	0.032	0.025	0.042	0.061	0.038		0.013
*S.com*	0.062	0.019	0.014	0.007	0.059	0.043	0.048	

*Note:* Distance values are shown below the diagonal, and the standard deviation above the diagonal.

Abbreviations: Bol, Bolivian origin; Bra, Brazilian origin; *S.com*, *S. compressus; S.elo*, *S. elongatus* ; *S.hol*, *S. hollandi* ; *S.mac*, *S. maculatus* ; *S.mar*, *S. marginatus* ; *S.mag*, *S. magallanesi*; *S.spi*, *S. spilopleura* ; *S.rho*, *S. rhombeus* .

### 3.3 Weight–Length Relationships

**FIGURE 5:**
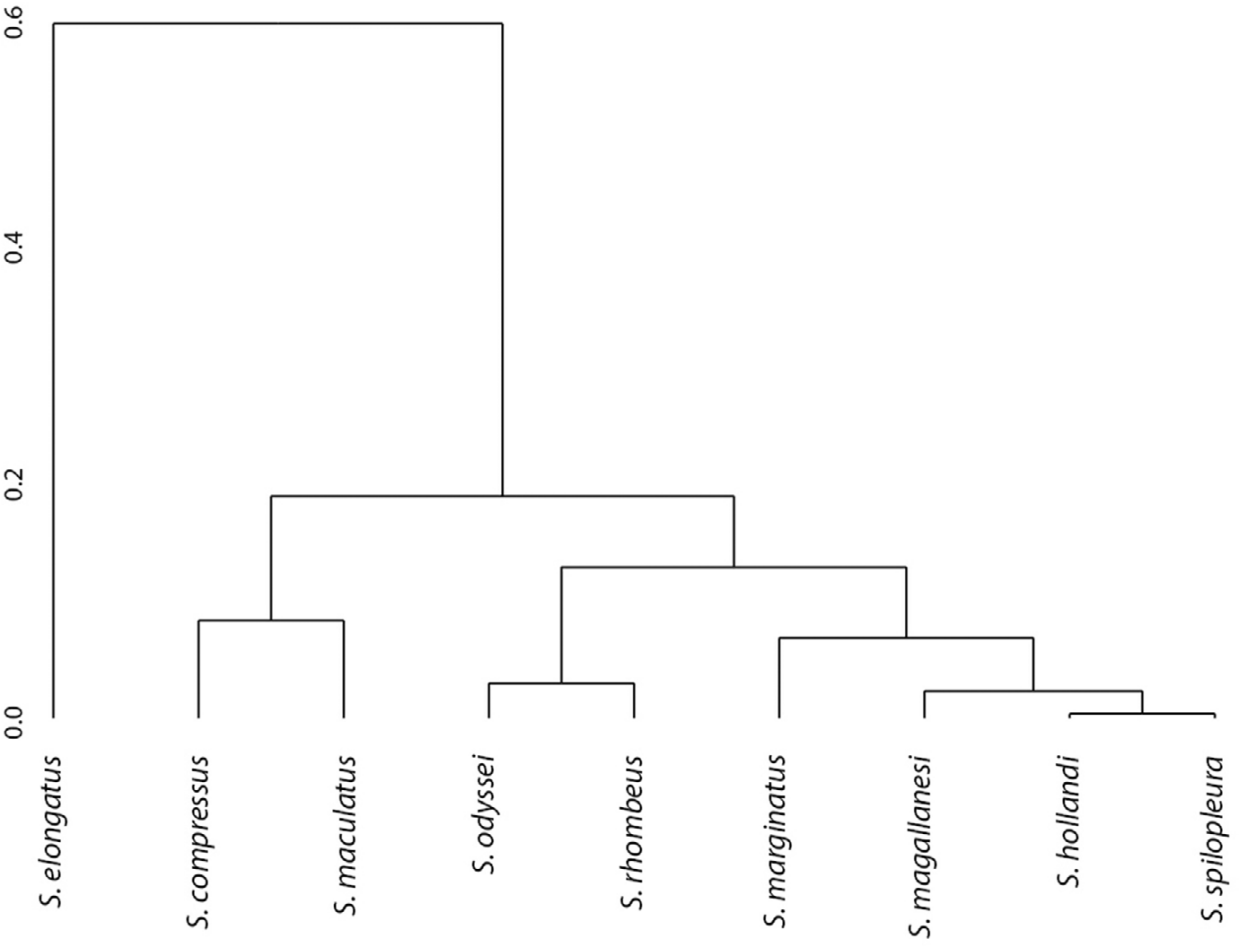
Hierarchical clustering of weight‐length relationship slopes among nine *Serrasalmus* piranha species from Bolivia. Euclidean distances, UPGMA method.

The phylogenetic linear mixed model showed a significant effect on weight by standard length (fixed variable, *Z*‐score = 89.74, *p* < 0.001), by species phylogenetic covariance (random factor, LRT = 5.18, *p* = 0.023), and by the random effect for the interaction between standard length and species (LRT = 4.27, *p* = 0.039), indicating that the phylogenetic signal was influencing the relationship between weight and standard length of the studied *Serrasalmus* species. The fixed effect model showed the same results, with significant differences among species (*F*_9, 206_ = 35.9, *p* < 0.001), and a significant interaction between standard length and species (*F*_8, 206_ = 2.5, *p* = 0.034). Pairwise comparisons of WLR slopes between species, showed that *S. elongatus, S. compressus, S. maculatus* and *S. marginatus* were the species that differentiated most from the rest (Table 3), and this was also evident from the hierarchical cluster analysis, where those four species were the most distant, with *S. elongatus* situated in a group of its own, *S. compressus* and *S. maculatus* grouped together as the second most differentiated group, and *S. marginatus* as an independent branch of the third group (Figure 5). A fourth group with *S. odyssei* and *S. rhombeus* was also formed. The most similar species, according to their WLRs, were *S. hollandi* and *S. spilopleura*.

**TABLE 3:** Triangular matrix of pairwise comparisons (Tukey adjustment) for the slopes of weight‐length relationships among nine piranha *Serrasalmus* species from Bolivia.

	*S.com*	*S.elo*	*S.hol*	*S.mac*	*S.mar*	*S.ody*	*S.rho*	*S.mag*	*S.spi*
*S.spi*	0.09	−0.60	−0.00	0.18	−0.06	−0.12	−0.16	0.02	
*S.mag*	0.07	−0.62	−0.02	0.15	0.08	0.15	0.18		0.27
*S.rho*	0.25	−0.44	0.15	0.33	0.09	0.03		0.35	0.23
*S.ody*	0.22	−0.47	0.12	0.30	0.06		**< 0.001**	0.07	0.99
*S.mar*	0.16	−0.53	0.06	0.24		0.57	**0.013**	**0.001**	**0.007**
*S.mac*	−0.08	−0.77	−0.18		**0.021**	**< 0.001**	0.08	**0.008**	**0.033**
*S.hol*	0.10	−0.59		**0.046**	**0.011**	0.11	0.69	0.57	0.26
*S.elo*	0.69		0.78	**0.04**	**< 0.001**	**0.005**	0.94	0.39	0.20
*S.com*		0.76	0.99	**0.019**	**0.004**	**0.02**	0.67	0.53	0.27

*Note:* Euclidean distances are in the superior triangular matrix, while *p*‐values of the comparisons are in the inferior. All significant *p*‐values are shown in bold.

Abbreviations: *S.spi*: *S. spilopleura* ; *S.rho*: *S. rhombeus* ; *S.mar*: *S. marginatus* ; *S.mag*: *S. magallanesi*; *S.mac*: *S. maculatus* ; *S.elo*: *S. elongatus* ; *S.hol*: *S. hollandi* ; *S.com*: *S. compressus* .

### 3.4 Genetic Divergence, WLRs, and MSL Differences

**FIGURE 6:**
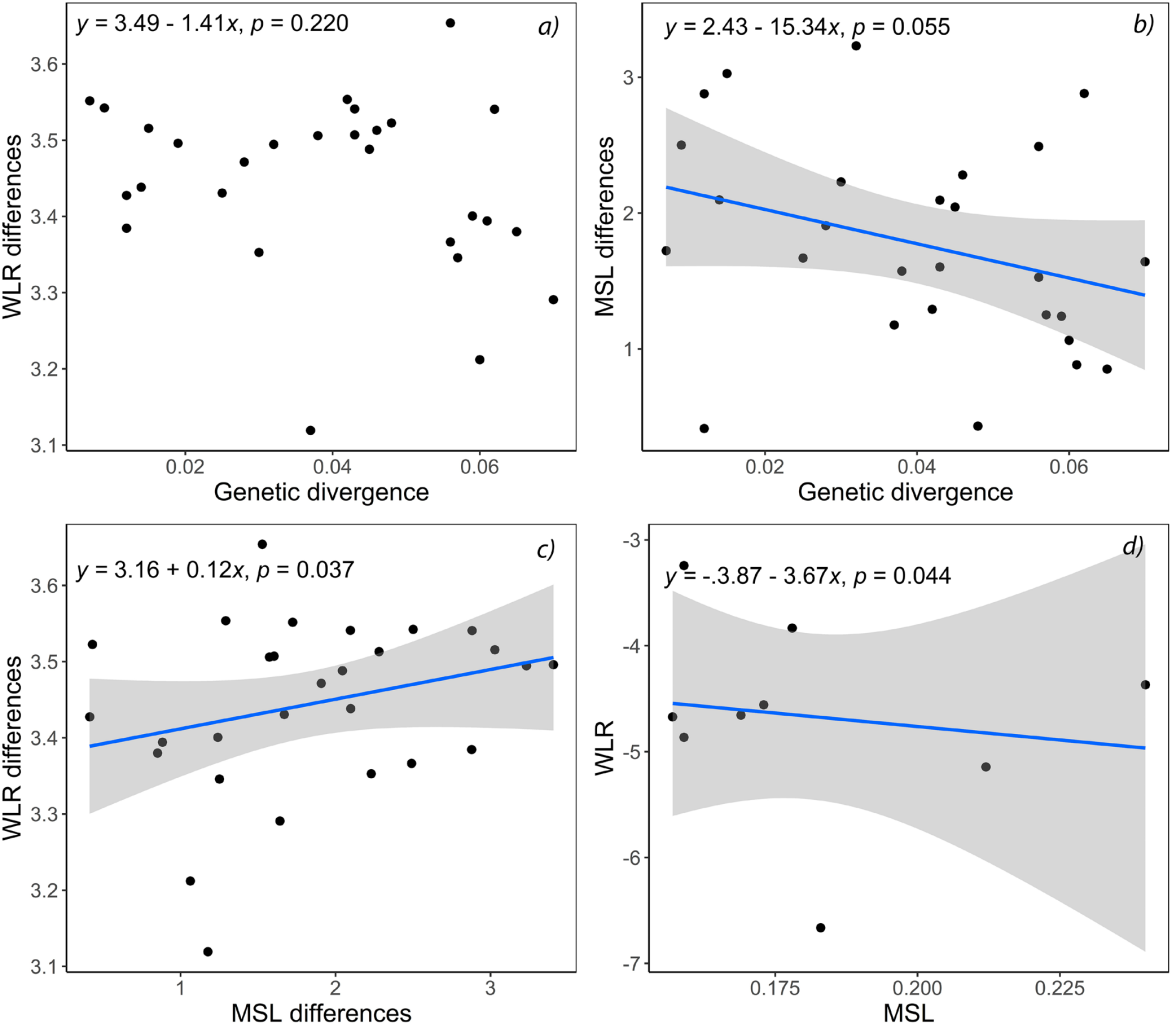
(a) Relationship between WLRs slope differences vs. genetic divergence of piranha *Serrasalmus* species from Bolivia. (b) Relationship between MSL differences vs. genetic divergence of piranha *Serrasalmus* species from Bolivia. (c) Relationship between WLRs slope differences vs. MSL differences of piranha *Serrasalmus* species from Bolivia. (d) Relationship between WLRs slope vs. MSL of each piranha *Serrasalmus* species from Bolivia. Standard deviation shown (in gray) for significant regression lines. All differences (WLRs, MSL) are expressed as Euclidean distances.

No significant relationship between the genetic divergence and the WLR differences between species (*t* = −0.39, *p* = 0.7, Figure 6a) was observed. There were, however, a marginal but non‐significant relationship between the genetic divergence and the MSL (*F*_1, 26_ = 4.02, *p* = 0.055, Figure 6b), and a significant positive relationship between WLRs and MSL differences of piranha species (*F*_1, 26_ = 6.14, *p* = 0.037), Figure 6c. Finally, the relationship between WLRs and MSL was negative (*F*_1, 26_ = 5.78, *p* = 0.044), showing that species that achieve larger SLs tend to gain weight slower than species with smaller sizes (Figure 6d).

## 4 Discussion

Growth pattern was variable among the nine species of piranhas studied from the Bolivian and La Plata basins. Coefficient *b* values showed that most of the piranha species have positive allometric growth. This denotes that as piranhas increase in length their body shape becomes rounder, and they grow faster in weight than in length. However, negative allometric growth was observed in *S. marginatus* denoting this species grows faster in length than in weight. Approximate isometric growth (proportional increase in weight and length) was observed only in the species *S. maculatus*. Coefficient value less than three in the former species suggests that juveniles of this species could be in a better nutritional stage (Froese 2006), but this could also be due to unsuitable environmental conditions influencing body condition in the species (Atama et al. 2013). We also recorded an extreme *b* value for *S. spilopleura* , but this is most likely caused by the small sample size (eight individuals) (Carlander 1985).

Both the genetic analyses and the WLRs relationships showed that *S. elongatus* was the most different *Serrasalmus* species in Bolivia. However, the rest of the *Serrasalmus* relationships shown by the genetic analyses on one hand, and the WLRs on the other, are not as clear nor coincide as much as with *S. elongatus* . Although in the following paragraphs we pinpoint the similarities and differences between the relationships shown by both analyses and attempt to present an ecological reason for those, it is convenient to note that our results showed a significant phylogenetic signal, indicating a probable dependence of WLRs on phylogenetic relationships in the Bolivian *Serrasalmus*. While we are aware that a phylogenetic signal does not always mean that evolutionary processes are involved (Revell et al. 2008), our results of WLRs and the ecological setup of Bolivian *Serrasalmus* suggest that they reflect a Brownian evolution model (i.e., random changes on the trait value) according to the environment. Furthermore, as most of these species have evolved on the Brazilian shield, which is known by its environmental/geological stability for fishes (Buckup 2011), it is possible that an environment‐bounded Brownian evolution (Boucher and Démery 2016) is taking place in this phylogenetic group.

Aside from *S. elongatus*, the piranha *S. maculatus* was genetically the most distant (differentiated) from the other species, but this genetic divergence was not concordant with the WLRs analyses. *Serrasalmus maculatus* is easily recognized by its robust head and jaws, rhomboidal body shape, yellow coloration, and a subterminal vertical dark band on the caudal fin. It is commonly caught alongside other piranha species near vegetation, at the bottom, and in open waters (FMCV pers. obs.). Its feeding habits appear to differ from those of other species, as *S. maculatus* appears to have fin‐nipping of other fish prey as one of the primary components of its diet (da Silva et al. 2015). The significant genetic divergence of this species may be linked to an ancient event driven by its specialized fin‐nipping feeding habits. However, although the genetic and ecological‐habits differentiation of this species is clear, our WLRs analyses fail to show differences between *S. maculatus* and *S. compressus* , grouping both species together. While the reason for this remains unclear, this pattern is possible under random (i.e., Brownian) evolution processes.

The genetic information also grouped the species *S. marginatus* , *S. magallanesi*, and *S. compressus*, with short length branches, denoting genetic proximity among them. Although genetically close, WLRs slopes were significantly different between *S. marginatus* and the other two species. This seems to stem from an early vicariant event, likely driven by changes in the hydrographic configuration and the recent separation of the La Plata (Bolivian special distribution for *S. marginatus* ) and Amazon drainages in certain areas of western Bolivia, on the plains of the Brazilian Shield craton (Koslowsky 1895; Lundberg et al. 1998; Wilkinson et al. 2006; Hubert et al. 2007; Latrubesse et al. 2012). Additionally, a close relationship between *S. spilopleura* and *S. hollandi* was observed based on both genetic data and WLR analysis. The close relationship observed between *S. spilopleura* and *S. hollandi* in both WLR slope and genetic variation in this study, as well as in previous genetic studies (e.g., Hubert et al. 2006, where *S. hollandi* was identified as *S. eigenmanni* ), and morphological traits (FMCV pers. obs.), suggests that they may be the same species, at least within the Upper Madeira system (Bolivian Amazon). This observation needs further study and verification.

In general, concordances between genetic and WLRs analyses could be related to some ancient (Friedman et al. 2020) or recent events of divergence in sympatry related to environmental conditions (Dikou 2023). As most of the species inhabit the same water bodies (i.e., lagoons, streams, rivers) in sympatry, a niche partitioning and/or seasonality (i.e., feeding, trophic plasticity, lateral migration) could be allowing the coexistence of species in the Bolivian Amazon basin. Something similar has been suggested for floodplain lagoons and rivers in Brazil (Neves et al. 2021; De Andrade et al. 2024). On the other hand, discordances observed between WLRs and genetic information could be influenced by intermediate processes of divergence (Hubert et al. 2008; Gallo‐Cardozo et al. 2024) or the randomness of evolutionary processes. Species delimitation in Bolivian piranhas appears to be more evident at the morphological level than the molecular level, likely due to an early stage of diversification of some lineages in the Amazon and La Plata basins (Gallo‐Cardozo et al. 2024). Differentiation in body size and shape (i.e., body elongation) is a repeated pattern of morphological variation in fishes and is influenced by habitat conditions and swimming mechanisms (DeLorenzo et al. 2023), because body size and shape dictate how fishes navigate their ecological niches, and often is associated with divergence along the benthic‐pelagic axis (Burns and Sidlauskas 2019; De Andrade et al. 2024). Enhanced swimming performance is especially advantageous in complex, highly structured environments, aiding in both predator evasion and prey capture (Langerhans 2009; Schrank et al. 1999). For instance, the elongated body of *S. elongatus* likely reflects an ancient divergence resulting from its adaptation to vegetated shore habitats. The elongated form of *S. elongatus* likely provides advantages for foraging within the dense floating or rooted vegetation along the shores of lagoons or streams, where small fish, macroinvertebrates (i.e., insects), and decapods are more abundant. In contrast, the more rounded species, such as *S. rhombeus* and *S. magallanesi* among others, are common in the open waters of lagoons and slow‐moving rivers or streams, where they can easily find their fish prey.

When considering the differences of MSL between species, it was shown that those were negatively related to genetic divergence, suggesting that when genetic divergence is greater, differences in MSL are smaller. Smaller MSL could indicate convergence in sympatric species (i.e., ecological but no morphological differentiation in response to selection caused by interspecific competition), which can facilitate the persistence of similar species in a diverse community as observed in the genus *Etheostoma* (Knouft 2003). Although only two out of nine *Etheostoma* species showed convergence in MSL influenced by the number of congeners, it is possible that *Serrasalmus* species with similar MLS can coexist within the highly diverse communities of the tropics. The complexity of community interactions is amplified, making excessive niche overlap unlikely. Low divergence among larger MSL and related species could also be governed by introgression observed among some species (Hubert et al. 2008), or an early process of speciation as has been suggested for some species of *Serrasalmus* in Bolivia (Gallo‐Cardozo et al. 2024).

The positive relationship between WLRs slope and MSL differences, as well as the negative relationship observed between WLR slopes and MSL, suggest that species with larger maximum size tend to increase in body mass more than in length. As in many vertebrates, piranha species probably originated at a relatively small size as compared with the range of sizes observed currently (Laurin 2004), although fossil records revealed that ancient piranhas could be larger than most species inhabiting lowlands of the Neotropical region (Cione et al. 2009). A higher increase in body mass than in length (as our results suggest) could be considered a good predictor of swimming capacity and metabolic traits in species (Rubio‐Gracia et al. 2020). Larger species can perform longitudinal migrations through turbulent rivers, which is more difficult for smaller species. The latter, which preferably perform horizontal migrations toward lentic and predictable habitats, such as lateral or meandric oxbow lagoons.

In summary, our study shows that phylogenetic relationships are still partially shown in WLRs of Bolivian piranhas, which coexist in sympatry (apart from *S. marginatus* ). It is probable that this group is still in the early stages of speciation, with ecological processes helping with niche differentiation and allowing *Serrasalmus* species coexistence. While WLRs do not fully reflect genetic distance or mtDNA COI phylogeny, they can be a useful measure to study evolutive processes and the environment effect on these.

## Author Contributions

**Fernando M. Carvajal‐Vallejos:** conceptualization (lead), data curation (equal), formal analysis (equal), investigation (lead), methodology (lead), project administration (lead), resources (lead), software (equal), supervision (lead), validation (lead), visualization (equal), writing – original draft (lead), writing – review and editing (lead). **Flavio Gallo‐Cardozo:** conceptualization (equal), data curation (lead), formal analysis (lead), funding acquisition (equal), investigation (equal), methodology (equal), project administration (equal), resources (equal), software (lead), supervision (equal), validation (equal), visualization (lead), writing – original draft (equal), writing – review and editing (equal). **Matías Careaga:** conceptualization (equal), data curation (equal), formal analysis (equal), funding acquisition (equal), investigation (equal), methodology (equal), project administration (equal), resources (lead), software (equal), supervision (equal), validation (equal), visualization (lead), writing – original draft (equal), writing – review and editing (equal). **Melina Campero:** conceptualization (equal), data curation (equal), formal analysis (lead), funding acquisition (equal), investigation (equal), methodology (equal), project administration (equal), resources (equal), software (lead), supervision (equal), validation (equal), visualization (equal), writing – original draft (equal), writing – review and editing (equal).

## Conflicts of Interest

The authors declare no conflicts of interest.

## Supporting information


Data S1.

## Data Availability

The data that support the findings of this study are available in the Supporting Information of this article.
